# Impact of Proximity to an Academic Health Center on Adherence to the National Comprehensive Cancer Network (NCCN) Guidelines in Patients With Soft Tissue Sarcomas

**DOI:** 10.7759/cureus.93647

**Published:** 2025-10-01

**Authors:** Zachary Butler, Dylan J Riley, Matthew Crosse, Steven Gitelis, Alan T Blank

**Affiliations:** 1 Orthopedic Oncology, Rush University Medical Center, Chicago, USA

**Keywords:** guideline adherence, nccn guidelines, proximity, soft tissue sarcoma, travel distance, treatment compliance

## Abstract

Background

Soft tissue sarcomas (STS) are rare and complex malignancies. Due to their infrequency and complexity, the National Comprehensive Cancer Network (NCCN) provides evidence-based guidelines to guide the workup and treatment of STS. Adherence to these guidelines is crucial, as demonstrated by studies showing improved survival outcomes with compliance. However, factors influencing adherence, such as proximity to treatment centers, remain underexplored. This study aims to assess adherence to the NCCN guidelines at a single academic institution in 2023 and identify factors that affect compliance.

Materials and methods

We performed a retrospective review of patients diagnosed with or treated for STS at a single academic institution in 2023. In accordance with NCCN guidelines and based on tumor staging, we reviewed whether patients received the proper workup and treatment, including a history and physical examination, primary site imaging, core needle biopsy, chest/abdominal imaging, presentation to a multidisciplinary team, wide resection surgery, radiation therapy, and chemotherapy.

Results

A total of 43 patients were included in the study. The most common diagnoses were spindle cell sarcoma and undifferentiated pleomorphic sarcoma, each occurring in 10 patients (22.73% each). The most common TNM stage was IV in 22 patients (51.16%). All 43 patients (100%) met workup compliance according to the NCCN guidelines. Thirty-four patients (79.07%) completed their treatment course, adhering to the NCCN guidelines throughout. Two (4.65%) underwent a complete workup but were seen as second opinions and returned to their previous provider to complete their care. Three (6.98%) were recommended for further treatment of either radiation therapy or chemotherapy but declined. These cases had travel distances of 1.5, 10.9, and 22.5 miles, respectively. Four (9.30%) passed away while undergoing treatment. Travel distance ranged from 1.5 to 201 miles, with a median distance of 31 miles and a median travel time of 55 minutes.

Conclusions

Our findings demonstrate a high rate of compliance with NCCN guidelines at a single academic institution. Contrary to expectations, proximity to the cancer center did not significantly affect adherence. Further studies are needed to explore additional factors influencing non-compliance, such as socioeconomic and healthcare system barriers.

## Introduction

Soft tissue sarcomas (STS) are a relatively rare malignancy, with approximately 13,590 new cases diagnosed annually in the United States, accounting for roughly 1% of adult cancers [1,2]. These tumors arise from mesenchymal cells and encompass a diverse group of neoplasms with varying behaviors and outcomes [3,4]. Due to their rarity and the complexity of management, the National Comprehensive Cancer Network (NCCN) has developed evidence-based guidelines to standardize the workup and treatment of STS, aiming to optimize patient outcomes and ensure high-quality care [5,6].

Adherence to these guidelines has been shown to significantly impact patient survival [7-12]. Voss et al. demonstrated that compliance with NCCN recommendations, particularly in the administration of radiation therapy, is associated with improved overall survival in patients with STS [13]. In their retrospective cohort study of 2,249 patients from the SEER cancer registry, individuals who received care in accordance with NCCN guidelines had better cancer-specific survival rates. This demonstrates the importance of following standardized treatment protocols in managing STS. Previous research has demonstrated the significant role that socioeconomic status (SES) plays in treatment adherence across cancer care. Specifically, studies have shown that lower income and education levels correlate with decreased adherence to recommended follow-up and treatment plans [14,15].

Despite the established benefits of guideline adherence, factors influencing compliance remain underexplored. Dunlop et al. highlighted that patients of lower SES were less likely to receive NCCN guideline-recommended radiation therapy, which correlated with poorer survival outcomes [4]. This finding suggests that socioeconomic disparities may play a critical role in access to and delivery of optimal cancer care. Along with this, Chen et al. highlighted racial disparities in receiving NCCN guideline-adherent care for ovarian cancer [16]. However, to our knowledge, there is limited research investigating other potential barriers to adherence, such as geographic proximity to treatment centers.

This study aims to assess adherence to NCCN guidelines for STS at a single academic institution throughout the year 2023. By evaluating compliance rates and identifying factors that may disrupt adherence, including patient proximity to the cancer center, we seek to gain a deeper understanding of the challenges in delivering guideline-concordant care. Our findings could inform strategies to improve adherence, thereby enhancing patient outcomes for those with STS.

## Materials and methods

Following approval from the Rush University Medical Center Institutional Review Board (approval number: 24072902), a natural language search was used to identify patients diagnosed with or treated for STS at Rush University Medical Center, a single academic institution, between January 1 and December 31, 2023. A total of 91 patients were identified through this search, representing all patients with STS records within the electronic medical record system during this period. Individuals were included if they received a diagnosis or underwent the majority of their workup or treatment during this period. The inclusion criteria encompassed all STS diagnoses, except for those involving the abdomen. These criteria were based on the diagnoses outlined in the NCCN Guidelines for STS of the extremity, body wall, and head/neck (version 1.2024) [5]. According to these guidelines, the standard evaluation for STS includes a comprehensive history and physical examination, imaging of the primary tumor site, core needle biopsy, staging imaging of the chest and abdomen as indicated, and review by a multidisciplinary sarcoma team. Treatment recommended per the guidelines consists of wide-margin surgical resection, with radiation therapy and/or chemotherapy considered based on histology, tumor size, and stage. After establishing our cohort, we then reviewed each patient's workup and treatment plan to evaluate adherence to these NCCN guidelines.

Next, the estimated travel time for each patient was calculated using Google Maps (Google LLC, Mountain View, CA, USA) in drive mode [17]. The endpoint for all calculations was our central downtown academic cancer center, where the majority of treatments are administered, with each patient's home address serving as the starting point. Travel times were standardized by using a consistent time and day (Tuesday departing at 0900 CST). This methodology was chosen to provide a standardized measure of commute burden and minimize variability in travel estimates due to day-to-day traffic fluctuations, holidays, or unusual weather events. This selected time was chosen to represent a typical commute time, excluding extreme rush-hour conditions, to ensure consistency in the estimates. Travel distances were also recorded using the shortest distance available.

Demographic and clinical data were analyzed using descriptive statistics. Continuous variables were reported as means with standard deviations (SD) or as medians with interquartile ranges (IQR), depending on the normality of the distribution. Comparisons between groups, such as patients whose treatment adhered to the NCCN guidelines versus those who did not, were performed using Student’s t-test or the Mann-Whitney U test, as appropriate. Categorical variables were reported as frequencies and percentages. Statistical significance was defined as a two-sided p-value of <0.05.

## Results

A total of 43 patients met the inclusion criteria for this study, which was conducted between January and December 2023. The overall cohort consisted of 25 males (58.14%) and 18 females (41.86%). Patients were predominantly White individuals (65.12%), followed by Black individuals (23.26%), with the remaining 11.63% reported as “other.” The cohort had a median BMI of 28.51 (IQR 24.28-31.06) with an average age of 60.12 ± 18.8 years. The most common diagnoses were spindle cell sarcoma and undifferentiated pleomorphic sarcoma, each accounting for 22.73% of cases (Table 1). Stage IV disease was the most common, affecting 22 patients (51.16%) of the cohort (Table 2).

**Table 1:** Breakdown of STS diagnoses RLE: right lower extremity, STS: soft tissue sarcoma

Diagnosis	Total	Percent (%)
Spindle cell sarcoma	10	23
Undifferentiated pleomorphic sarcoma	10	23
Liposarcoma	5	11
High-grade STS	2	5
Rhabdomyosarcoma	2	5
Clear cell sarcoma	2	5
Myxofibrosarcoma	2	5
Ewing sarcoma (soft tissue variant)	2	5
Synovial sarcoma	1	2
Leiomyosarcoma	1	2
Fibrosarcoma	1	2
Low-grade STS	1	2
Myxoid liposarcoma	1	2
Myofibroblastic sarcoma	1	2
Epithelioid sarcoma	1	2
Adenocarcinoma metastatic to RLE soft tissue	1	2

**Table 2:** Breakdown of TNM staging TNM: tumor, node, and metastasis

TNM staging	Total	Percent (%)
IA	1	2
IB	1	2
IIA	4	9
IIB	3	7
IIIA	6	14
IIIB	6	14
IV	22	51

All 43 patients (100%) met full compliance with the NCCN guidelines for workup, which included a thorough history and physical examination, primary site imaging, core needle biopsy, chest/abdominal imaging, and presentation to the multidisciplinary team. Thirty-four (79.07%) patients completed their treatment course at our institution, adhering to the NCCN guidelines throughout. Four (9.30%) passed away while undergoing treatment. Two (4.65%) did not continue treatment after seeking a second opinion and returning to their original healthcare provider; adherence to NCCN guidelines in these patients could not be independently verified. Three (6.98%) were recommended for further treatment, such as radiation therapy or chemotherapy, but declined. In total, 35 (81.40%) patients underwent wide-margin surgical resection. Thirty-four (79.07%) received radiation therapy, and 27 (62.79%) received chemotherapy.

The travel distance to the cancer center ranged from 1.5 to 201 miles, with a median distance of 31 miles (IQR, 17.5-55). Travel times ranged from eight minutes to three hours and 20 minutes, with a median time of 55 minutes (IQR, 40-72.5 minutes). There was no significant difference in compliance rates between longer travel distances (p = 0.209). The three cases of treatment non-compliance, in which patients declined further treatment, had travel distances of 1.5, 10.9, and 22.5 miles.

## Discussion

This study aimed to assess adherence to NCCN guidelines for STS in 2023 at a single academic institution that treats approximately 200 new sarcoma patients annually. This study also aimed to investigate factors influencing compliance, such as patient proximity to the health center. Overall, 100% of patients met workup compliance, demonstrating a high level of adherence to the standards outlined by the NCCN for optimal care in STS management. However, nine patients (20.93%) failed to comply with treatment for various reasons. Unfortunately, four (9.30%) passed away while receiving treatment. Three (6.98%) were recommended for further treatment, such as radiation therapy or chemotherapy, and declined. Additionally, two (4.65%) were seen as second opinions and returned to their original provider for treatment after obtaining a proper workup.

Interestingly, contrary to our hypothesis, distance from the cancer center did not appear to be a significant factor in adherence to guidelines. The median travel distance was 31 miles, with a range from 1.5 to 201 miles. The three cases in which individuals declined further treatment traveled relatively short distances of 1.5 miles (eight minutes), 10.9 miles (35 minutes), and 22.5 miles (55 minutes), suggesting that factors other than geographic distance may be more influential in determining adherence. This finding aligns with research by Voss et al., who identified SES as a more significant determinant of compliance, particularly in accessing radiation therapy, than geographic proximity [13].

Previous literature has highlighted the importance of compliance with NCCN guidelines in improving outcomes for patients with STS. Voss et al. demonstrated that strict adherence to the guidelines, including proper staging, imaging, and treatment protocols, correlates with improved survival outcomes [13]. Other studies, such as those by Dunlop et al., emphasize the challenges that patients from lower socioeconomic backgrounds face in accessing necessary treatments, particularly radiation therapy, which can lead to worse cancer-specific survival [4]. While our study did not show a significant association between proximity and NCCN guideline adherence, other factors, such as SES barriers, likely play a crucial role. Research on adherence to cancer care has identified that lower income and education levels, along with limited insurance coverage, are significant determinants of non-compliance, especially in underserved populations [14,15].

Several limitations should be noted in this study. This is a retrospective study with a relatively small sample size, limited to a single institution. We chose this cohort and specific diagnosis for uniformity, and we realized it is challenging to find significant differences in outcomes among groups with very high compliance rates. Additionally, while travel distance was explored as a factor in guideline adherence, other variables such as SES, insurance coverage, and patient comorbidities were not analyzed. They may provide further insight into the barriers to optimal care. Furthermore, Google Maps provides an estimated travel time, while actual travel times may vary due to external factors such as accidents or construction. Finally, as the endpoint of the distance traveled was our central downtown academic cancer center, our institution has numerous satellite treatment centers, which may have helped with adherence to NCCN guidelines.

## Conclusions

This study demonstrates a high level of compliance with NCCN guidelines for extremity STS at a single academic institution. Distance to the central academic cancer center did not appear to be a significant factor in adherence. Future studies should investigate additional variables, including socioeconomic and healthcare system factors, to gain a deeper understanding of the full scope of influences on guideline adherence in sarcoma care.
